# The Eukaryotic Microbiome: Origins and Implications for Fetal and Neonatal Life

**DOI:** 10.3389/fped.2016.00096

**Published:** 2016-09-09

**Authors:** William B. Miller

**Affiliations:** ^1^Independent Researcher, Previously affiliated with Pinnacle Health, Harrisburg, PA, USA

**Keywords:** microbiome, hologenome, holobiont, metagenome, epigenetics, niche construction, neonatal

## Abstract

All eukaryotic organisms are holobionts representing complex collaborations between the entire microbiome of each eukaryote and its innate cells. These linked constituencies form complex localized and interlocking ecologies in which the specific microbial constituents and their relative abundance differ substantially according to age and environmental exposures. Rapid advances in microbiology and genetic research techniques have uncovered a significant previous underestimate of the extent of that microbial contribution and its metabolic and developmental impact on holobionts. Therefore, a re-calibration of the neonatal period is suggested as a transitional phase in development that includes the acquisition of consequential collaborative microbial life from extensive environmental influences. These co-dependent, symbiotic relationships formed in the fetal and neonatal stages extend into adulthood and even across generations.

## Introduction

In only a relatively few years, two complementary lines of research have intersected to dramatically alter our perceptions of the organizational structure of eukaryotic macroorganisms. The first of these is our enlarged understanding of the actual composition of all macroorganisms beyond the organic singularities that had been traditionally presumed. It is now apparent that all macroorganisms are hologenomic entities. Such organisms represent vast collaborations of mutually competitive and co-dependent cellular ecologies that entwine highly diverse microbial constituencies with innate eukaryotic cells (1–3).

The second insight is our developing appreciation of the exact expanse of this co-aligned microbial realm. Our prior understanding was based on microbial culturing techniques and has been widely expanded through contemporary genomic research and metagenomic analysis (4). This analysis has illuminated the astounding depth and variety of the microbial sphere with which all multicellular eukaryotes are linked (5). It is estimated that fewer than 10% of microbes can be cultured, and that may yet be an overestimate (6). This missing fraction was revealed by the analysis of 16S rRNA gene sequence amplification that disclosed completely unanticipated microbial lineages (7). Metagenomic techniques were developed to extend research beyond phylogenetic descriptions of microbial community associations. This has provided deeper insight into genetic trees, physiological mechanisms and has yielded the discovery of a number of novel genes and details of nutrient cycling (8). As a result, there is a new understanding of the commonality of horizontal genetic transfers (9–13). All of these contemporary findings have significant repercussions for eukaryotic growth, individual development, and health throughout the life cycle. Consequently, the fetal and neonatal microbiomes can be reappraised within this new frame and pregnancy can be viewed as fetal-maternal co-development. As initial background toward that reassessment, the origins and development of the eukaryotic microbiome will be reviewed, indicating an enlarged perspective as to its significance: the eukaryotic microbiome extends beyond symbiotic appendage and merits consideration as a full player in eukaryotic life. The experiential forces that contribute to the origins and development of the fetal and neonatal microbiome will then be outlined within that context. It will be presented that the fetal and neonatal periods represent critical stages of microbial aggregation and combination that have a substantial impact on health of the developing infant, its future well-being, and that of future generations.

## Holobionts: An Essential Partnership

It is now apparent that all multicellular eukaryotes are holobionts (1, 3). There are no exceptions. The extensive linkages between eukaryotic cells and their microbial partners in localized tissue ecologies maintain metabolism, the immune system, and general balance of health that sustain eukaryotic macroorganisms. Until recently, our general regard of the microbial sphere was principally one of combat. Instead, our truer narrative is one of collaboration, co-linkage, and co-dependency as well as more obvious competition. Growing evidence suggests that reciprocal signaling between eukaryotic cells and their collaborating microbial partners significantly influences normal development of the eukaryotic individual (1, 14, 15). Therefore, the neonatal period must be considered beyond any mere coordinated innate series of ontological and physiological neurohumoral phases toward an enlarged perception. Each developmental stage of any life cycle across its entire arc is now understood to be dependent on microbial contributions that continually shift with the macroorganism. The neonatal period is a meaningful stage for the acquisition and deployment of vital microbial partnerships, both as a part of a larger inter-generational developmental arc and a significant component of well-being throughout the remainder of the life cycle.

Therefore, a proper understanding of the neonatal period requires a reappraisal of the circumstances of macroorganic life. Current estimates suggest that there are at least 100 trillion microbes that are in and on us, including bacteria, viruses, and fungi. In total, they easily outnumber our primary cells by a factor of 10 to one or more (4, 5). The entirety of the genetic complement of this associated microbiome vastly outnumbers our own innate one (16). Although there has been a movement toward some revision of those raw numbers (17), the conclusions about the nature of eukaryotic multicellular organisms as functional holobionts remains steady.

Current research is informing us of the truer extent of the intimate dependencies shared between our microbial partners and ourselves. In view of these interrelationships, some have considered eukaryotes as multi-species units (2) or “super-organisms” (18). Indeed, it is no longer tenable to regard eukaryotic macroorganism as any inherent singularity. Instead, a more accurate perspective is that multicellular eukaryotic organisms remain firmly rooted within their inherent cellular nature. As vast collaborative enterprises of co-linked, cooperative, co-dependent, and competitive ecologies, these merge together so coherently as to seem one discrete entity. Therefore, any traditional concept of “host” and “guest” no longer strictly applies across this seamless developmental diachronic arc (3). This contemporary conceptualization of multicellular eukaryotic organisms displaces the historical notion of innate “us” and conjoined microbial life as “other” into a modern appraisal of an organic entity that is a consensual “we.” All evolutionary development of all eukaryotic macroorganisms is derived from unicellular roots and remains perpetually anchored within cellular processes and conditions (19–23). Consequently, growth and development of all macroorganisms can now be appropriately viewed within a firmly cellular trajectory. Therefore, the neonatal period can be represented as one crucial stage of temporal variation of these mixed cellular ecologies that must perforce exist within immunological rules (3).

## The Microbiome in Health and Disease

Current research is revealing that our reproductive potential, developmental stages, immune system, and metabolism are a coalescence of both innate cellular and microbial traits (4, 14). The microbiome is the vast numbers and varieties of microbes, their genes, and their metabolites that are embedded features of all mucosal surfaces, digestive tract, skin, and all bodily tissues. Each assemblage plays a critical role for the optimal function of our gut (24), brain and central nervous systems (25, 26), respiratory (27) and immune systems (28, 29), and oral cavity (30).

Our understanding of the range of influences of the microbial sphere in each of these cellular ecologies as well as their extensive networks of interconnections continues to enlarge. The gut microbiome is a critical determinant of both innate immunity and adaptive immune systems of all eukaryotic macroorganisms at all stages of life (31). For example, intestinal dysbiosis with a breakdown of homeostatic balance of a healthy gut microflora has been linked to inflammatory bowel disease and susceptibility to enteropathogens (32, 33). Significant gut dysbiosis has also been linked to metabolic syndrome and obesity (34), and can have a profound influence on the CNS (26). Studies have demonstrated that changes in the gut microbiome can substantially alter brain function and behavior through neural, endocrine, and immune pathways. In a similar manner, the respiratory system is now understood to have its own intricate ecological microbiome, analogous to the gut microbiome, whose equilibrium must be maintained to resist bacterial overgrowth and the development of respiratory infections (35).

It has been estimated that at least 30% of mammalian metabolites have a bacterial origin. Approximately 37% of human genes have with homologs in Bacteria and Archaea of which 28% are estimated to have originated in unicellular eukaryotes (15). The intrinsic metabolic drive of all complex organisms is supported by a variety of microbial participants. One crucial aspect of these specific microbial balances within each tissue ecology is the protection against pathogens that grants survival. For example, bifidobacteria provide protection in the gut from enteropathogenic infection through production of acetate (36). These interrelationships are so overlapping and co-dependent that any individual eukaryotic organism must be viewed as intertwined ecological co-dependent cellular habitats. In this complex association, fundamental processes of community ecology apply, such as dispersion of nutrients and constituents, degree of variation and specialization, or selection pressures (28).

## Variations within the Human Microbiome

### Characteristics of the Microbiome among Species and Individuals

Research has demonstrated that microbiomes remain similar within species though subject to wide variation of the exact mix and match (37). Nevertheless, surveys of human gut microbial ecologies suggest that these microbiomes can be pertinently classified within broad “enterotypes” based on the varied abundance of an expanse of microbial constituents derivative of ancient mammalian microbiomes (38). This would not be surprising since the inheritance of bacterial DNA and bacteria by epigenetic mechanisms are now well established (39). Further yet, the impact of the virome is just as significant (40), and is now understood to be a consequential component of human ecosystems, including its evolutionary development (41). For example, LTR class I endogenous retrovirus (ERV) retroelements, a distant relative of HIV, has considerably impacted the transcriptional network of human tumor suppressor protein p53 (42). This is a master gene regulator crucial for primate differentiation. Therefore, retroelements are one significant component of the evolutionary development of the regulatory network of transcription factors in a species-specific manner.

Recent investigations have been directed toward identifying a general core human microbiome. As such, the concept of a minimal obligatory gut metagenome in functional terms is now regarded as a feature of human life and is a consequential aspect of its health and development (43). Metagenomic sequencing of the human gut has demonstrated a microbial gene set that is more than 150 times larger than the innate human gene cohort. At least 1000 bacterial strains have been identifiable, which are largely shared among all humans (44). A survey of 18 body sites in over 200 individuals has demonstrated general stability of the microbiome among greater than 95% of participants (45). Interestingly, it was the vaginal microbiome that demonstrated the least commonality. Even within that localized variation, the dominant microbial contribution was the *Lactobacillus* genus with a considerable variation of sub-genus types. In general, Huse et al. have found that the human microbiome sticks to type, even with respect to the neonatal birthing experience (45).

While it now seems to be true that the microbiota has general stability among individuals of the same species, an integrated survey of its spatial and temporal distribution demonstrates significant complexity. In any given individual, the microbiota is personalized and has significant spatial variability across body habitats (46). Furthermore, systematic temporal variation has also been revealed. It seems likely that the pace of this temporal variation, though life long, is greatest from the fetal state extending across infancy.

### Temporal Variation of the Fetal and Neonatal Microbiome

Research is now indicating that the fetal stage has a substantial influence on the microbiome of the neonate and beyond. Although it has long been believed that the uterus, amniotic fluid, and the fetus are sterile, this has been an incorrect assumption. It is now apparent that the placenta harbors a unique microbiome. This is characterized by non-pathogenic commensal microbiota from the Firmicutes, Tenericutes, Proteobacteria, Bacteroidetes, and Fusobacteria phyla that most closely resembles the maternal oral microbiome (47). The ramification has been the demonstration of *in utero* colonization of the infant gut (48, 49). Microbes can be identified in meconium, predominantly belonging to the Enterobacteriaceae family along with lactic acid bacteria, including Leuconostoc, Enterococcus, and Lactococcus (48, 50). This microbial profile strongly resembles that of young infants, but importantly is significantly influenced by a range of maternal factors. For example, maternal eczema has been linked to newborn respiratory disorders (51). Therefore, there is a necessary overlap of the maternal immune system with that of the fetus and this extends beyond the direct innate maternal mechanisms that were previously assumed to be the only operative ones.

The ongoing development and maturation of the neonatal gut microbiota, and presumably then every other localized microbiome of any neonate, is affected by numerous factors that represent a developmental arc extending from mother to child. This includes maternal diet or weight gain, probiotic use, pre-natal, peri-natal, or post-natal antibiotic use, mode of delivery, or feeding regimen (52). These influences can be significant and last across childhood and also carry the potential to affect the entire adult life cycle. For example, C-section delivery altering the normal exposure to vaginal microbiome has been associated with an increased risk of celiac disease, Type 1 diabetes, asthma, and obesity (53). Maternal antibiotic use has been linked to an 84% increase in childhood obesity and a significant increase in asthma that can continue into adulthood (53).

Through profiles of vaginal and milk samples, maternal and paternal stool samples, and metagenomic samples from siblings, it has been demonstrated that microbial communities vary widely from baby to baby based on all sources. There are considerable temporal variations, each with distinct features, that range across weeks to months (54). However, by the end of the first year, most of these idiosyncratic microbial ecologies tended to converge toward a characteristic adult profile in a non-random pattern of succession (55). Even given those changes, the infant at 11 months remains its own individual with phylotypes that can be distinct from the mother (56). A strong resemblance of a typical adult gut microbiome does not ensue until 2–3 years of age with specific assemblages demonstrating geographically based population differences (57).

Mode of delivery and feeding regimen appear to have large initial influences on this variation. The highest incidence of advantaged infant gut microbial ecology (defined as the highest counts of bifidobacteria and lowest numbers of *Clostridium difficile* and *Escherichia coli*) among term infants born vaginally and exclusively breast-fed (58). Vaginal delivery promotes colonization by maternal vaginal and fecal bacteria (*Lactobacillus, Bacteroides, Fusobacteria*), whereas infants born by cesarean section have a greater number of microbes associated with skin and the hospital environment (*Staphylococcus, Corynebacterium, Propionibacterium*) (48, 59). Comparisons between breast or formula-fed infants show larger populations of *Bifidobacterium* and *Lactobacillus* among those that were breast-fed and greater numbers of *C. difficile, Bacteroides, Streptococcus*, and *Veillonella* among the formula-fed cohort (48). These microbial shifts may account for the association of impaired development of the neonatal immune system and altered metabolic parameters later in life when infant formula-feeding is substituted for breast-feeding (53).

## Heritable Aspects of the Human Microbiome

Over the last two decades, there has been a critical reappraisal of previously discredited Lamarckism toward an increasing acceptance of the impact of the heritable nature of acquired characteristics (60–62). Such heritable overlaps between parent and child are much more complex than previously imagined. Sampling of the placenta and amniotic fluid demonstrates bacteria representative of the maternal gut and oral microbiomes whose mechanism of transfer is unknown; it is postulated that the transmission travels via lymphatic networks for the gut (63) or the blood stream secondary to gingival inflammation (64). Although the heritable transmission of symbionts has been known to occur in invertebrates for over 50 years, it had remained controversial in humans and other mammals until recently (49). The mechanisms of transfer are both internal and external and the mechanisms are not always clear in either case (1, 2). For example, breast milk, though long thought sterile, actually has hundreds of bacterial strains that vary over the course of lactation (65, 66).

Such transfers are now believed to carry a range of consequences that are greater than any simple sharing of microbial types. It is now contended that there is sufficient developmental and phenotypic plasticity to permit an organism to adjust its phenotype and that of its offspring in direct response to the environment (67, 68). The heritable microbiome is part of that reciprocating system. Heredity extends beyond parental genes to encompass the heritable transmission of extensive developmental resources between parent and offspring. The trajectory of an organism is thereby shaped by adjustments by both internal and external influences along developmental paths.

A better understanding of this type of interplay can be gained through the concept of niche construction theory. This holds that an organism is not only influenced by its environment but also reciprocally influences it. For example, in the macro sphere, earthworms chemically alter the soil, but in so doing, also provide themselves with an environment in which their renal function is optimally suited. In this manner, they improve their own fitness and then indirectly provide a similar service to other proximate species (69). Adaptation is also a reciprocating process, in which an organism participates in ameliorating some of its selection pressures within local environments. Therefore, it can be represented that this reverberating interchange also occurs between the microbial sphere and our innate cellular composition in a continuous enactment of mutual niche construction at a cellular level, as a mirror image of similar processes in the macro environment. This reciprocating interaction is an important aspect of the development of any organism and extends across our life span. Yet, it is reasonable to suppose that it is most consequential during the developmental phases of any macroorganism. As such, the full range of development of any organism can now be better understood as a product of a combination of discrete heredity, its collaborating microbial consort, environmental impacts, and heritable genetic transfers. Each of these is always in reciprocation with the others and the outward environment. This perspective empowers a new understanding of ourselves as a continuous process of organism–environmental complementarity that extends across our life spans and even beyond.

Within such a framework, Gilbert proposes a “re-tell” of the human birth narrative. In these terms, birth is the origin of a new community (70). It is not merely the eukaryotic body that is being reproduced, but a set of overlapping and complex microbial/eukaryotic cellular symbiotic relationships. Therefore, pregnancy may be due, in part, to these symbiotic interrelationships in which the fetus has its own reciprocating microbial community. The immune mechanisms between mother and fetus are thereby influenced and modulated by a set of countervailing responses from the entire microbiological cohort that participates.

## The Impact of the Microbiome on Development

All of these factors permit the reappraisal of the neonatal period. It can be viewed as the reciprocating intersection of an overarching maternal influence on the dynamics of the multi-source accretion of a neonatal microbiome that is significant for its development. In essence, pregnancy and birth are co-development with interlocking niche construction and the “passage from one set of symbiotic relationships to another” (70) (p.1). It is suggested that the best means through which this might be conceptualized is a form of “host” scaffolding in which microbial constituents colonize a eukaryotic organism. Chiu and Gilbert reinforce this same point (18). They argue that the relationship between humans as holobionts and our essential microbial fraction is an instance of reciprocal scaffolding, developmental mutualism, and ecological niche construction. In such circumstances, mother and fetus and their respective symbionts constitute the conditions for the development and reproduction of the other. These reciprocal relationships include the direct induction of both maternal and neonatal physiological changes so that ecological relationships within varying tissues are changed. Crucially, since this process begins before birth and then continues thereafter, it is not surprising that changes in the maternal microbiome are now being linked to a variety of differing pregnancy outcomes and pre-term birth (71).

When neonatal development is considered within such an enlarged holobionic frame, the widespread and enduring influence of epiphenomena impacting mixed cellular/microbial ecologies in full-term infants can be better understood. Persistent cognitive abnormalities in malnourished children are linked, in part, to a persistent pattern of immature gut microbiota; specific regions of the brain exhibit persistent neotenous patterns of gene expression associated with later deficits in higher cognitive functions. It is believed that the brain has been made vulnerable by gut dysbiosis induced by malnutrition (72). In this manner, it is suggested that the development of the microbiome in the neonatal period and infancy should be considered as a developing “microbial organ.” The gut flora is recognized as having a collective metabolic activity equal to a “virtual organ within an organ” (73). It also becomes clear that the development of that “organ” is a function of a significant overlap with the maternal microbiome. For example, Gosalbes et al. used culture-independent genetic screening to demonstrate a high incidence of maternally transferred antibiotic-resistant genes in infant’s meconium and fecal samples, at least some of which is presumed to have occurred *in utero* (51).

Therefore, the critical aspect of the microbial cohort of every holobiont must now be considered in explicit developmental terms along its long arc of life, beginning before conception. Multiple tissue ecologies function both at an individual level and collectively as part of a combined set of coordinating mixed cellular/microbial constituencies that comprise any eukaryotic multicellular organism. In such circumstances, it is implicit that any such understanding must fully appraise the overlapping sources of all superimposed localizing environmental influences of all types (67, 68).

## New Perspectives on the Neonatal Microbiome

Since all eukaryotic multicellular organisms are holobionts, our viewpoint of development can now be re-framed. The complex collaborative partnerships that enable all multicellular eukaryotic life exist across an entire developmental landscape that is inherently enacted at the microscopic level rather than the more easily assessed macroscopic one (3). As Gilbert points out, “Some material in the mother’s milk is for the bacteria and not the infant.” (70) (p.5). Maternal milk sugars intersect with the newborn immune system in one stage of its developmental arc to enable the successful reproduction of a particular set of bacteria. In reciprocity, that bacterial strain and others can then enable the diachronic unfolding of developmental capacities of the infant.

Given these overlaps, some have suggested that a mental picture of a core microbiome might be likened to a Venn diagram with overlapping circles that indicate the membership of a sample within human habitats (74). Yet, any such diagram misses a vital point. Holobionts are flux agencies in which ecological partnerships vary over time as a crucial shifting balance between innate cells and a variable as well as an obligatory co-aligned microbiome.

These complexities point to the probability of a long journey of discovery about these necessary co-dependencies. It is now acknowledged that the outward universe cannot now be properly framed outside of the existence of dark matter that has only recently been validated. Our genomes cannot be currently understood absent the “junk” DNA that is no longer considered such (75–78). Similarly then, it is likely that the boundaries between what has been termed facultative microbial life and that which is currently defined as obligate will also undergo very substantial modification in the future (79, 80). It is likely that the meaning of commensal, mutualist, and symbiont will evolve over time. In fact, as our understanding enlarges, a change in terminology may be required to reflect these consequential differences. Commensal bacteria are recognized mutualists that supply a eukaryote with essential nutrients by assisting in the metabolism of otherwise indigestible compounds. Importantly too, they form a constituency that unites with innate cells, such as gut mucosa, to defend against super-infections by potential pathogens, such as C*. difficile* (81). Yet, the difference between a “good” microbe and a “bad” one can become blurred; some types of normal gut bacteria can become pathogenic, in which they are termed a “pathobiont” (29). In such circumstances, it is not easy to sort with any exactitude the means by which intestinal dysbiosis leads to dysfunction of the adaptive immune system or inflammatory bowel disease. The complexity of these interrelationships is so great that Round and Mazmanian (29) have even raised the possibility that the mammalian immune system is controlled by microorganisms and has not been devised to control them as always presumed.

## Intervention in the Neonatal Microbiome

There are many advantages to reconsider the exact circumstances of the neonatal period. A fuller perspective of the conjoined and overlapping developmental arcs of the maternal and fetal microbiomes improves opportunities for intervention. Studies of the microbial status of the placentas of pre-term infants predict white matter damage and later cerebral palsy (82). Certain prenatal placental microbial patterns that are specifically associated with infection from maternal skin microflora presage echolucent lesions, ventriculomegaly, and diparetic cerebral palsy. Furthermore, it appears that the pre-term infant gut microbiota is different from that of term infants. Pre-term infants demonstrate a much larger inter-individual variation than among healthy full-term infants, thereby suggesting an opportunity for protective adjustment (83). For example, although no specific pathogen has been identified as a direct causal factor in necrotizing enterocolitis (NEC), a low diversity of specific gut microbial constituencies has been determined in pre-term infants with NEC compared to full-term controls (84). In infants likely to go on to NEC, a substantial increase in Proteobacteria and a decrease in Firmicuties within the first week of life was detected (85). Importantly, it seems that pre-natal influences are consequential in this general dysbiosis. The result has been considered a dysmature response to microbial colonization with a resultant breach of the intestinal epithelial barrier involving a breakdown of intestinal immune homeostasis (86, 87).

The potential of these types of interactions is far-ranging. Reciprocating developmental relationships form a rich gut–brain axis that provides potent linkages between neonatal sepsis and NEC with long-term psycho-motor disabilities (88). Attempts to ameliorate this are already underway. There are now several interventions being studied in pre-term low birth weight infants through the prophylactic administration of specialized prebiotics and probiotics. This approach seems to offer a tantalizing hope that gut microbial dysbiosis is amenable to manipulation and a decrease in the incidence if NEC (89, 90).

Nor is the neonatal gut, the only microbiome of consequence. Microbial dysbiosis in the neonatal lung includes aspects similar to that of the gut and has shown an association with the incidence of bronchopulmonary dysplasia (BPD). A reduced diversity of the neonatal lung microbiome may be a significant factor in the subsequent development of BPD that is independent of immune mediators and cytokine levels (91). Furthermore, research is demonstrating a highly complex and little-understood interplay between the development of neonatal gastrointestinal and respiratory microbiota and the subsequent regulation of immune function (92). Similarly, the neonatal and infant skin microbiome has only recently been explored in any detail. It is now known that the skin microbiome is transiently influenced by mode of delivery but evolves rapidly over the first year of life, demonstrating steadily increasing microbial diversity (93). The exact role of this temporal variation of the evolving infant skin microbiome in protection against pathogens or the modulation of the immune system is yet unclear though certainly important. It is possible that the skin microbiome directly influences the correct development of the epidermis after birth (94). Recent research has demonstrated that the microbiome extends below the epidermal layer, again emphasizing the presence of active microbial colonization and ecological interplay at a site that had always been presumed sterile (95).

Therefore, properly adjudicating this passage can be expected to have substantial impacts on neonatal health that extends into childhood and adult life. Disturbance of the acquisition of any desirable microbial fraction, either from maternal influence or mode of delivery (96), can now be linked from this transitional phase to subsequent diverse childhood and adult outcomes that encompass allergic status (97, 98) and obesity (99). For example, investigations have demonstrated possible associations between maternal or neonatal administration of antibiotics and the subsequent development of childhood and adult allergies and asthma (100–102).

There are other aspects of life-long repercussions of cellular/microbial relationships that would have been considered remote until recently. Microbial partnerships mediate the physiology of all eukaryotic organisms and actively participate in a wide range of neurodevelopmental capacities. Experiments with mice have demonstrated diminished motor activity and an increase in anxiety-like behavior when the gut microbiota is suppressed (26). This is associated with altered gene expression patterns and appears to involve messaging pathways that affect neuronal circuits. The microbiota–gut–brain axis, as an emerging concept, has been linked to altered brain development and behavior, neurodevelopmental disorders, and autism (103). Experiments with gut-microbiota-suppressed mice demonstrate significant social impairment. It is apparent that microbiota has a role in the programing of normal species social behaviors. A fuller understanding of these gut–brain interrelationships is now indicating a role for the microbiota as an unconscious system regulating behavior. Such impacts have been seen on cognitive function, stress management, and diverse neurodevelopmental patterns, such as autism spectrum disorders (104, 105). Therefore, therapeutic manipulation of the enteric microbiome is actively being investigated (106). Furthermore, increasing research into epigenetic mechanisms indicates the substantial potential for these types of consequences to reverberate back and forth between generations in complex skeins not imagined until only a very few years ago (11–13).

Although it is clear that the neonatal period is necessary for the acquisition, succession, and development of a robust microbial fraction of hologenomic life, there is never any explicit endpoint while living. Even in adulthood, the spectrum continually shifts, subject to epiphenomena, such as antibiotic treatments, dysbiosis, obesity, and environmental impacts. Indeed, the opposing end of the life spectrum, a consistently shifting “elderly” microbiome may have its own consequential impact reciprocal to our understanding of the temporal variation that characterizes infancy (107).

Indeed, the essential consideration is that the very concept of host, obligate mutualist, opportunistic symbiont, parasite, or pathogen must be entirely re-explored across an entire life cycle. A requirement for optimal intervention then becomes the detailed understanding of all the specifics of the human microbiome. This is particularly so for the neonatal period in which continuous epigenetic changes and shifts are occurring in both obligate and facultative microbial assemblages in continuous reciprocity with the environment (108).

It is now becoming increasingly evident that substantial progress toward the successful treatment of many diseases will be dependent upon a much deeper understanding of the temporal relationships of the human microbiome. This seems especially the case for many chronic afflictions, such as inflammatory bowel disease, obesity, and some mood disorders (109–111). That complexity can begin to be unraveled by emerging sequencing capacities and computational technologies. There is a reciprocal side to any such exploration. A full understanding of human health requires an appreciation of a life-long arc of individual temporal variation of a personalized microbiome that represents a continuum of individual proclivities, cultural milieu, and parentage. Any in-depth understanding of the neonatal period and its developmental processes and metabolic status requires an assessment of foods, medications, and individual immunological capacity, including adaptive experiences of both child and parent. This becomes an appraisal of a full panoply of pertinent information beyond the traditional scope. It would be a happy occurrence if all these shifts of any minimal microbiome might yield direct and simple analysis toward treatments. Yet, that is likely not. It is becoming ever clearer that idiosyncratic characteristics of the entirety of all holobionts both unite us as a species but still separate us from all others like ourselves. The future of medicine will need to accommodate this complex individuality.

It is pertinent to note that progress toward that specific goal will need a much greater degree of standardization of the techniques for sample gathering and analysis of the microbiome than prior studies. These have been understandably constrained by the difficulties inherent in obtaining and sampling microbial communities in any tissue ecology in a reproducible manner so that results can be fruitfully compared. Recent research studies have attempted to address these sources of error (112, 113). In these reports, stool samples were immediately frozen by the participants and picked up by investigators instead of being sent in by mail. Consequently, there was a high level of concurrence between the analytic findings in both of these studies. Additionally, a very broad range of markers and environmental factors was considered, including known diseases, drug usage, multiple smoking categories, and extensive dietary factors. Such meticulous techniques will be a requirement going forward. However, even this will represent a continued significant limitation until it can be applied beyond the bacterial domain with similar scrupulous analysis extended into associated viral and fungal constituencies.

Even given these variables, there will be substantial progress. The contemporary realization that our modern metabolism has evolved via serial exaptations from cellular roots has an increasingly direct bearing on current medical practice. It will also explicitly pertain to future productive research in human physiology, metabolism, and immune function (20, 22, 23). It is a cellular world, dominated by cellular imperatives, reiterated in local and shared cellular ecologies to enact macroorganisms that to our superficial appraisal are singular but decidedly, are not. Therefore, the applicable drama ever and always dwells within a cellular realm dominated by immunological rules by which eukaryotes actually live (3).

## Conclusion: A Vast Frontier

Intersecting recent discoveries confirm that there is an arc of associated microbial and innate cellular interactions that engage as holobionts and extend beyond any traditional age-related touchstones of intrinsic neurohumoral development. Within this frame, each eukaryotic macroorganism is now known as a complex collaboration of mixed cellular and microbial ecologies constituted by the entirety of its innate cells and its associated and shifting microbiome. Therefore, our understanding of development must incorporate an unfolding narrative of microbial aggregation and disassembly in concert with our innate cells. Collectively, these elaborate the metabolic stream that promotes development and governs our life cycle. This naturally extends across the attainment of reproductive ability and proceeds then inevitably to senescence and death. Within this entire arc, the neonatal period is an active crossroad. It is within this temporal phase that critical interrelationships are formed and it can be asserted that these ever govern that arc. They begin with invisible threads even prior to the zygotic stage, are nurtured and adjudicated in the womb, and extend beyond individual life itself as either epigenetic shadow or privilege in the generations beyond.

The manner in which the neonate meets and accommodates this interplay will in many respects settle the future of the adult organism (114). Plainly then, a major frontier in neonatology is the far-reaching and still little explored interstices of this arc of conjoined life. As properly appraised, the neonatal period becomes a crucial stepping stone toward either future advantage or debility, … ever and always, for better or worse, securely anchored to both its immediate and ancient past.

## Glossary

Microbiome – The complete set of microorganisms in a particular environment.

Hologenome – The sum of the genetic information of the innate cells of an organism and its entire associated microbiome.

Holobiont – A macroorganism representing a combination of its innate cells and entire microbiome, i.e., all multicellular eukaryotic macroorganisms.

Metagenome – All the genetic material present in an environmental sample.

Epigenetics – Heritable changes in gene expression not due to changes in the underlying DNA code. Anything other than innate DNA sequences that influences the development of an organism.

Niche construction – The process by which an organism interacts with and alters environments, generally in a manner that increases its chances of survival.

## Author Contributions

The author confirms being the sole contributor of this work and approved it for publication.

## Conflict of Interest Statement

The author declares that the research was conducted in the absence of any commercial or financial relationships that could be construed as a potential conflict of interest.
